# Current Practices for Reference Gene Selection in RT-qPCR of Aspergillus: Outlook and Recommendations for the Future

**DOI:** 10.3390/genes12070960

**Published:** 2021-06-24

**Authors:** Meagan Archer, Jianping Xu

**Affiliations:** Biology Department, McMaster University, 1280 Main St. W, Hamilton, ON L8S 4K1, Canada; archermm@mcmaster.ca

**Keywords:** reference gene, *Aspergillus*, RT-qPCR, validation, aflatoxin biosynthesis, antifungal resistance, actin, beta-tubulin, 18S rRNA, glyceraldehyde 3-phosphate dehydrogenase

## Abstract

*Aspergillus* is a genus of filamentous fungi with vast geographic and ecological distributions. Species within this genus are clinically, agriculturally and biotechnologically relevant, leading to increasing interest in elucidating gene expression dynamics of key metabolic and physiological processes. Reverse-transcription quantitative Polymerase Chain Reaction (RT-qPCR) is a sensitive and specific method of quantifying gene expression. A crucial step for comparing RT-qPCR results between strains and experimental conditions is normalisation to experimentally validated reference gene(s). In this review, we provide a critical analysis of current reference gene selection and validation practices for RT-qPCR gene expression analyses of *Aspergillus*. Of 90 primary research articles obtained through our PubMed query, 17 experimentally validated the reference gene(s) used. Twenty reference genes were used across the 90 studies, with beta-tubulin being the most used reference gene, followed by actin, 18S rRNA and glyceraldehyde 3-phosphate dehydrogenase. Sixteen of the 90 studies used multiple reference genes for normalisation. Failing to experimentally validate the stability of reference genes can lead to conflicting results, as was the case for four studies. Overall, our review highlights the need to experimentally validate reference genes in RT-qPCR studies of *Aspergillus*.

## 1. Introduction

The ascomycete genus *Aspergillus* is among the first described filamentous fungi, dating back to 1729, as recorded by Pier Antonio Micheli, an Italian biologist and priest. Under a microscope, the asexual spore-forming structure of these fungi looks like an aspergillum, a “holy water sprinkler”, and Micheli named these fungi in the genus *Aspergillus* [1,2]. Since then, over 300 species have been described and recognised in this genus [3]. These species differ in a diversity of morphological, physiological and phylogenetic characters. *Aspergillus* fungi are broadly distributed across the globe, and are found in diverse ecological niches such as soil, composts, water, buildings, air, and in or on plants [4]. Species in this genus have significant impacts on many fields, including biotechnology (e.g., antibiotics production) [5], fermented food production [6], food safety (e.g., mycotoxin production and food contamination) [7] and human health [2]. In addition, several *Aspergillus* species have been model organisms for understanding the fundamental biology, including physiology and genetics, of fungi and eukaryotes [8,9].

An emerging theme on the studies of these organisms is the regulations of gene expressions and metabolic pathways, and how the regulated expressions are related to their beneficial and detrimental effects to human welfares. For example, several *Aspergillus* species are employed frequently in the food and beverage industry as fermenters of soy, to make sake, miso and soy sauce [6]. Understanding how the genes are involved in producing these products could help develop strategies to control their expressions for optimal commercial productions. On the other hand, several other *Aspergillus* species are opportunistic human fungal pathogens (HFPs), including *Aspergillus fumigatus*, *Aspergillus flavus*, *Aspergillus niger*, *Aspergillus terreus* and *Aspergillus nidulans* [10,11]. Globally, each year there are approximately 4.8 million cases of allergic bronchopulmonary aspergillosis (including asthma), 3 million cases of chronic pulmonary aspergillosis and 250,000 cases of invasive aspergillosis [12]. The dominant cause of aspergillosis in humans is *A. fumigatus* [10]. Furthermore, *A. flavus* can not only cause human infections, but along with *Aspergillus parasiticus*, can also produce aflatoxins that contaminate foods and severely impact human health, with long-term exposure leading to infertility and endocrine disorders [7].

Among human hosts infected with pathogenic *Aspergillus*, effective treatment often requires antifungal drugs such as itraconazole, voriconazole and amphotericin B [13]. Currently, voriconazole is the recommended first-line of treatment and prophylactic agent against invasive aspergillosis [14]. However, drug-resistant strains are increasing in both environmental and clinical populations of *A. fumigatus* and other opportunistic species [2]. Infections by drug-resistant fungal pathogens are associated with elevated length of hospitalisation and higher mortality [15]. Understanding the mechanism(s) of drug resistance in *Aspergillus* pathogens, including *A. fumigatus*, could help monitor and improve treatment options. In addition, there is increasing evidence that secreted enzymes in *A. fumigatus* play an important role in pathogen colonisation and host tissue damage. Intriguingly, strain Z5 of *A. fumigatus* has multiple xylanases [16], which can break down xylan into its constituents of xylose, arabinose and glucuronic acid, all of which can then be used in the production of biofuels [17]. Better control of the specific pathways involved in producing these beneficial enzymes could generate significant economic benefits.

Over the years, several approaches and techniques have been developed to monitor and quantify gene expression. These techniques include Northern blotting, microarray hybridisation, high throughput transcriptome sequencing and reverse-transcription quantitative polymerase chain reaction (RT-qPCR). RT-qPCR quantifies the amount of mRNA in a biological sample, and takes this as a measurement of gene expression [18,19]. RT-qPCR possesses several advantages over other methods: it is quick, capable of high-throughput processing, and is highly sensitive and specific [18,19]. Additionally, RT-qPCR is useful for detecting low-abundance transcripts, as the high annealing temperature used during RT-qPCR allows for highly specific primer binding to the target gene [20]. Indeed, RT-qPCR is often used to confirm the results obtained using other approaches [21,22,23,24,25,26,27,28,29,30,31,32]. However, in order to accurately quantify gene expression using RT-qPCR, the normalisation of mRNA levels to validated reference genes is required [18]. Through normalisation using appropriate reference genes, the impact of differences in RNA yield (due to variation in extraction), cDNA yield (due to variation in reverse-transcription) and amplification efficiency on gene expression levels can be minimised [19]. Thus, by controlling for these differences, normalisation allows for the comparison of mRNA levels across different experimental treatments [19].

A good reference gene is one that is stably expressed under the experimental conditions being tested [19]. The Minimum Information for publication of Quantitative real-time PCR Experiments (MIQE) guidelines recommend using two or more validated reference genes to normalise gene expression data [19]. Using the geometric mean of multiple reference genes achieves high accuracy of normalisation, and is recommended over the arithmetic mean, as it better controls for differences in the amount of mRNA and outliers between genes [33]. When using multiple reference genes, it is also important to select genes that are not co-regulated, as co-regulated genes may lead to false positives as they lead to stable expression ratios [33]. Overall, inappropriate and/or insufficient reference genes can lead to the wrong interpretation of results, and reduce the reliability of experimental data [18,19]. Therefore, careful consideration should go into the selection of reference genes for RT-qPCR analysis.

In this review, we describe the reference genes that have been used for normalising gene expressions in RT-qPCR analyses for species in the genus *Aspergillus*. We review how the reference genes were selected in these studies, and whether they were validated under the specific experimental conditions. In addition, we briefly summarise how these reference genes were used in RT-qPCR to help understand the important biological processes in these fungi. Towards the end, we discuss the potential areas of improvements for selecting robust reference genes.

## 2. Reference Genes for Gene Expression Analyses of *Aspergillus*

To assess the state of reference genes in RT-qPCR studies of *Aspergillus*, in this review, we searched PubMed using the search query: “Aspergillus qPCR”. The loose search criteria were used to assure that we cast a wide net for any relevant studies available for our review. In total, this query returned 575 results which were manually curated to exclude those used to quantify fungal load, or those that did not specifically examine gene expression in *Aspergillus*, but that mentioned members of the genus in the abstract and with RT-qPCR studies in organisms other than *Aspergillus*. This allowed us to obtain information from 90 primary studies (Table 1, Table S1) [16,21,22,23,24,25,26,27,28,29,30,31,32,34,35,36,37,38,39,40,41,42,43,44,45,46,47,48,49,50,51,52,53,54,55,56,57,58,59,60,61,62,63,64,65,66,67,68,69,70,71,72,73,74,75,76,77,78,79,80,81,82,83,84,85,86,87,88,89,90,91,92,93,94,95,96,97,98,99,100,101,102,103,104,105,106,107,108,109,110] and one reference gene validation study [111], from 2001 to 2020. For each of these 90 studies, we manually extracted information on the species, genes, experimental conditions, purpose of the study and analytical methods used.

**Figure 1:**
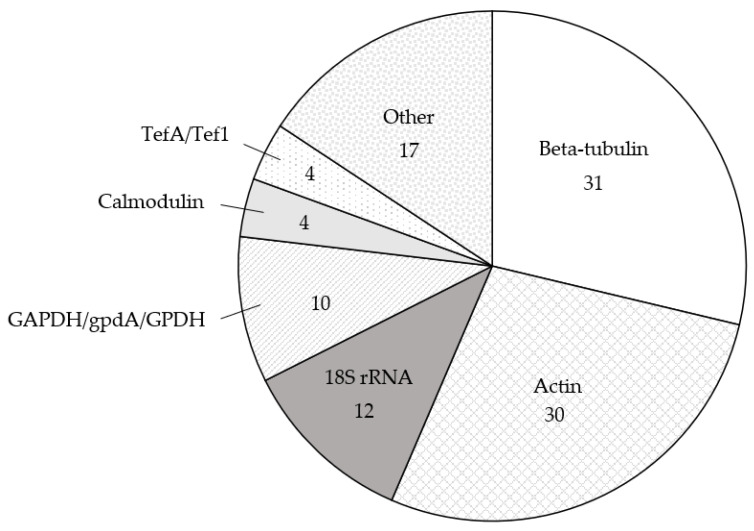
Distribution of reference gene usage across 90 RT-qPCR studies of *Aspergillus*. Note that in the 90 studies examined, reference genes were used a total of 108 times, as some studies used multiple reference genes. Therefore, 108 was used as the denominator when computing the frequency of reference gene usage. The group “other” is comprised of reference genes that were used fewer than four times. Beta-tubulin was the most frequently used reference genes, at 31 times in the literature.

Overall, 15 species of *Aspergillus* were examined: *A. aculeatus* [34,35]; *A. carbonarius* [32,36,37]; *A. cristatus* [24]; *A. fischeri* [38]; *A. flavus* [28,39,40,41,42,43,44,45,46,47,48,49,50,51,52,53,54,55,56,57]; *A. fumigatus* [16,21,22,23,27,31,58,59,60,61,62,63,64,65,66,67,68,69,70,71,72,73,74]; *A. luchuensis* [26,75]; *A. nidulans* [30,76,77,78,79,80,81,82,83,84,85,86]; *A. niger* [87,88,89,90,91,92,93,94]; *A. nomius* [95]; *A. oryzae* [25,83,88,96,97,98,99,100,101]; *A. parasiticus* [29,48,50,55,102,103,104,105,106]; *A. sojae* [102]; *A. terreus* [67,68,107,108]; and *A. westerdijkiae* [109,110] (Table 1, Table S1). The most common reference gene used in these studies was beta-tubulin, which was used 31 times in the literature [38,41,42,46,47,48,49,50,51,52,53,54,55,56,62,63,64,65,66,67,68,69,70,76,80,81,82,83,98,106,109], followed by actin (30 times) [16,21,22,23,24,25,26,30,34,42,43,44,45,60,61,75,76,77,78,79,89,90,91,96,97,103,104,105,107,108], the 18S ribosomal RNA gene (18S rRNA; 12 times) [27,28,29,36,39,40,41,58,59,87,88,102] and glyceraldehyde-3-phosphate (*GAPDH*, *gpdA* and *gpdh*; 10 times) [30,31,35,52,54,60,71,90,92,110] (Table 1, Figure 1, Table S1). Reference genes that were used four or fewer times [16,27,34,37,38,41,53,57,60,70,72,73,74,82,84,85,86,93,94,95,99,100,101] are grouped under Section 2.5 of this review.

In Section 2.1, Section 2.2, Section 2.3, Section 2.4 and Section 2.5 below, we briefly describe the history of use for each reference gene, as well as the relevant research results of these gene expression studies in *Aspergillus*. We also provide an analysis of the consistency or inconsistencies of the studies regarding the validation of reference genes. Validation of specific reference genes for use under the specific experimental conditions tested was provided in only 17 of the 90 studies (Table 1, Table S1) [16,27,30,32,35,38,41,46,52,54,55,77,86,94,103,105,107]. Table 1 is organised alphabetically first by species and second by reference gene, followed by the date of publication, and includes the species examined, reference gene used, whether the reference gene was validated and the experimental conditions for each of the 90 papers returned by our PubMed search. A brief explanation of how the reference genes were validated, the associated gene symbol for each reference gene, strains used, type of sample taken, number of biological replicates and normalisation strategy are described in supplementary Table S1. Together, our review highlights the need for more frequent reference gene validation and standardising normalisation practices in RT-qPCR gene expression studies of *Aspergillus*.

### 2.1. Beta-Tubulin

Beta-tubulin is a subunit of a universal eukaryotic protein called tubulin, the basic structural unit of microtubule. Tubulin is a heterodimer, consisting of equal numbers of alpha-tubulin and beta-tubulin subunits [112]. Microtubules are part of the cytoskeleton, and provide structure and shape to eukaryotic cells. Because of the essential role microtubules play, both the alpha- and beta- tubulin encoding genes are highly conserved; as a result, they are commonly used for evolutionary studies. For example, the beta-tubulin gene is commonly used in phylogenetic [113] and taxonomic [114] studies of fungi. However, the copy number of beta-tubulin in *Aspergillus* genomes can vary [115]. For example, there are two copies of beta-tubulin in *A. nidulans*, while there is only one copy in *A. niger* [115]. In *A. nidulans*, each beta-tubulin performs a different function. The *benA*-encoded beta-tubulin is involved in nuclear movement and vegetative growth during mitosis [112,116], while the *tubC*-encoded beta-tubulin is involved in conidiation [112].

Beta-tubulin has been used as a reference gene in gene expression studies since 1988 [117], and in RT-qPCR studies as early as 2000 [118]. In this review, beta-tubulin was used as a reference gene 31 times in studies examining gene expressions in *A. nidulans* (5) [76,80,81,82,83], *A. fumigatus* (9) [62,63,64,65,66,67,68,69,70], *A. flavus* (13) [41,42,46,47,48,49,50,51,52,53,54,55,56], *A. parasiticus* (4) [48,50,55,106], *A. oryzae* (2) [83,98], *A. terreus* (2) [67,68], *A. westerdijkiae* (1) [109] and *A. fischeri* (1) [38] (Table 1, Table S1). Of these 31 studies, nine provided a rationale for choosing beta-tubulin as a reference gene [38,41,46,47,52,55,62,98,109], though only six provided proper validated justification [38,41,46,52,54,55]. Many of these studies examined the impact of abiotic factors, such as the wavelength of light [82], nutrient availability [64,76,106], water activity [47], temperature [67,106] and pH [76] on the expressions of metabolic and biosynthesis genes. A select few examined the effects of biotic factors, such as the impact of co-incubation with *Streptomyces spp.* [42,54], *Eurotium cristatum* [56], *Debaryomyces hansenii* CYC 1244 [109] and *Listeria monocytogenes* [41] on the expressions of genes associated with aflatoxin biosynthesis. Several studies examined the effects of antifungal treatment on the expressions of antifungal resistance-associated genes [62,68,80], while others explored alternative treatments for minimising aflatoxin production [42,50,52,54,55,56]. Targeted and spontaneous mutations were also heavily examined in the literature, characterising the function of putative genes [65] or the specific effects on metabolic [38,66,81,83] and antifungal resistance [69] pathways. The following two sections will discuss the experimental conditions and findings of the studies that validated beta-tubulin as a stable reference gene for use under the specific conditions of the study, and those that did not provide justification for its use, respectively. Studies that specifically aimed to validate reference genes under a specific set of conditions will be discussed below in Section 3.

#### 2.1.1. Studies That Validated Beta-Tubulin Expression Stability under the Experimental Conditions Tested

Of the 31 studies that used beta-tubulin as a reference gene for RT-qPCR, only six validated the reference gene for use under the specific experimental conditions being studied [38,41,46,52,54,55]. Cacares et al. 2016 and Lappa et al. 2019 followed the “gold standard” for reference gene selection by using NormFinder [119] to evaluate the stability of multiple reference genes (of which beta-tubulin was one of them) in their studies examining the expression of genes related to aflatoxin biosynthesis [41,52]. In their analyses, beta-tubulin was demonstrated to be stably expressed during *A. flavus* growth under specific experimental conditions: at 27 °C on malt extract agar (MEA) supplemented with 0.5 mM eugenol [52]; and during growth in malt extract broth (MEB) at 25 °C and 30 °C in the presence or absence of *L. monocytogenes* [41]. In addition to validating stability, both research groups also used multiple reference genes for normalisation [41,52], thus applying the “gold standard” for RT-qPCR data normalisation [19].

Previous studies have identified that 30 genes are involved in aflatoxin biosynthesis in *A. flavus* [7]. These genes include the specific regulators *aflR* and *aflS*, and several early (*aflA*, *aflB*, and *aflC* or *pksA*) and later (*aflD* or *nor-1*, *aflM* or *ver-1*, *aflN*, *aflO*, *aflP* or *omtA*, and *aflQ*) pathway genes as well as those not shown to be directly associated with the pathway (*aflT*) [7]. Here we will use the naming conventions of the aflatoxin biosynthesis genes as recommended by Yu et al. [120]. Aside from these genes, several global regulatory genes were also identified to be associated with aflatoxin biosynthesis, including *veA* [121], *mtfA* [122] and *msnA* [123]. With this background information, here we briefly review the studies that investigated the potential influences of environmental factors on aflatoxin production through RT-qPCR.

In their 2016 study, Carces et al. found that 26 out of the 27 aflatoxin biosynthesis genes that they examined showed decreased expression following treatment with 0.5 mM eugenol, where the only gene that did not decrease in expression was *aflT* [52]. They also demonstrated that the genes involved in the later steps of the AFB_1_ biosynthesis pathway, such as *aflQ*, were more affected by eugenol treatment than early pathway genes, such as *aflA* and *aflB* [52]. Moreover, they found that global regulators *veA*, *mtfA* and *msnA* were upregulated [52].

In another study published by the same group two years later, Caceres et al. assessed the efficacy of *S. roseolus* as a biocontrol agent against AFB1 aflatoxin production in *A. flavus* [54]. The group used the same method of quantification for RT-qPCR as they did previously, and the same reference genes, including beta-tubulin [54]. They examined the expression of 27 genes in the aflatoxin biosynthesis pathway, as well as the impact of *S. roseolus* co-incubation on the expression of genes involved in fungal development, response to external stimuli and oxidative stress [54]. As was observed in their study investigating the effects of 0.5 mM eugenol treatment, twenty-six of the 27 genes in the AFB1 aflatoxin biosynthesis pathway showed significantly decreased expression after four days of co-incubation with *S. roseolus*, where again, only *aflT* expression was not significantly reduced [54]. Moreover, the expression of the two AFB1 transcriptional regulators, *aflR* and *aflS*, was significantly reduced 6-fold, and the genes involved in early aflatoxin biosynthesis were less affected than those involved in the intermediate and later stages [54]. This suggests that reduction in the expression of aflatoxin biosynthesis pathway genes may be mediated by a reduction in *alfR* and *aflS*, where the few AFlR-AFLS complexes translated were used up for the expression of the early pathway genes, leading to fewer complexes available for the expression of the late pathway genes, and consequently a greater reduction in their expression [52]. Since *aflT* does not possess the binding site for the AflR transcription factor, and is therefore not regulated by the AFLR-AFLS complex [124], this may explain why *aflT* expression is unaffected following either treatment with 0.5 mM eugenol [52], in their previous study, or by co-incubation with *S. roseoulus* [54]. Given that the results of the two studies are the same with regards to the expression pattern of the aflatoxin biosynthesis genes, the use of beta-tubulin (and GAPDH discussed below) is reasonable, despite the experimental conditions being different (treatment with 0.5 mM eugenol [52] versus co-incubation with *S. roseoulus*) [54]. Additionally, following co-incubation with *S. roseoulus*, several of the genes involved in fungal development increased in expression, with *mtfA* being the most affected global regulator, exhibiting a significant 3.5-fold increase in expression [54]. Of those involved in the fungal response to external stimuli, *gprA* was the most affected, with its expression significantly increasing 5.5-fold [54]. Of the ten oxidative stress genes investigated, the two most affected genes were *atfB* and *cat2*, which decreased 149.2-fold and increased 3.7-fold, respectively [54]. Altogether, co-incubation with *S. roseoulus* was effective at reducing AFB1 biosynthesis and protein concentration, making it an attractive alternative to phytopharmaceuticals used in agriculture [54].

Lappa et al. demonstrated that there was a temperature threshold for aflatoxin expression, and that *L. monocytogenes* decreased aflatoxin production at the protein level [41]. Mayer et al. also provided experimental evidence for stable beta-tubulin expression in their study monitoring *aflD* expression in *A. flavus* during growth on wheat in a petri dish for nine days [46]. They found that *aflD* mRNA levels were highest on the fourth day of incubation, and decreased after day six, the day at which AFB_1_ protein levels first became detectable [46]. These results demonstrated that there was a transient induction of *aflD* expression in *A. flavus* before translation of AFB_1_ begins [46]. Similarly, Devi and Sashidhar stated that the antimicrobial peptides (AMPs) used in their study did not affect the expression of the reference gene, beta-tubulin, although they found that each of the four AMPs tested (PPD1 (FRLHF); 66-10 (FRLKFH); 77-3 (FRLKFHF); and D4E1- 12 (FKLRAKIKVRLRAKIKL)) significantly reduced the expression of the aflatoxin biosynthesis genes *aflR*, *alfC*, *aflD*, *aflM* and *aflP* in *A. flavus* and *A. parasiticus* [55]. Moreover, of the four AMPs tested, two (77-3 and D4E1) were the most effective inhibitors of expression [55]. Together, these RT-qPCR studies using beta-tubulin as a reference gene helped reveal the general conditions associated with increases (e.g., two days of incubation with wheat), and decreases (e.g., eugenol and AMP treatments and co-incubation with *L. monocytogenes)* in aflatoxin production by *A. flavus* and *A. parasiticus.*

Aside from being used as a validated reference for monitoring the expression of genes involved in mycotoxin productions in *A. flavus*, beta-tubulin has also been used for studying the regulation of gene expression related to other phenotypes. Specifically, in their study examining the role of mannitol in conidia stress resistance and ascospore development, Wyatt and colleagues provided experimental evidence for the stability of beta-tubulin expression during *A. fischeri* growth on oatmeal agar for up to six days [38]. However, they found that the mRNA levels of beta-tubulin were low in 30-day ascospores, and thus chose to normalise beta-tubulin and target gene expression to an additional reference gene, experimentally validated histone 3 (*his-H3*), for 30-day culture data [38]. They measured the expression of *mpdA*, *mtdA*, *mtdA* and *esdC* in wildtype and in three *mpdA* deletion mutants [38]. *mpdA* was maximally expressed in 6-day ascospore-forming wildtype cultures, at which time *mtdA* and *mtdB* were also highly expressed, indicating that these genes may play a role in mannitol metabolism during early ascospore development [38].

#### 2.1.2. Studies Missing Proper Beta-Tubulin Expression Stability Validation

Among the studies that used beta-tubulin as a reference gene, 25 did not provide experimental validation for its uses under the specific conditions tested [42,47,48,49,50,51,53,56,62,63,64,65,66,67,68,69,70,76,80,81,82,83,98,106,109]. These studies included those examining the expression of clinically relevant genes, such as those contributing to antifungal resistance [62,63,68,69,80], mycotoxin production [109], aflatoxin biosynthesis [42,47,48,49,50,53,56,106] and infection in steroid and chemotherapeutic mouse models [70]. They also included studies investigating changes in the expression genes associated with nutrient-sensing [76], calcium transport [66], iron acquisition [64], germination and conidiation [82], as well as those with potential industrial applications [83,98]. Other studies focussed on characterising proteins, such as the heat shock protein, Hsp60 [67] and sialidase enzyme [65]. One study also looked at how targeted mutations to the Spt-Ada-Gcn5-acetyltransferase components of *A. nidulans* affected the expression of an aldehyde dehydrogenase and three alcohol dehydrogenases [81].

When reference gene expression stability is not validated, it can be difficult to interpret conflicting RT-qPCR results. For example, Abdel-Hadi et al. reported that 90_W_ water activity delayed the expression of the aflatoxin biosynthesis activator, *aflR*, and that the structural genes, *aflD*, *aflM* and *aflP*, were expressed prior to the detection of *aflR* mRNA [47]. Moreover, at 85_W_, only *aflD* and *aflM* were expressed [47]. This indicates that structural gene expression may be independent of *aflR* under these conditions [47], contrary to the findings of Degola et al., who found that expression of the structural genes *aflD*, *aflO* and *aflQ*, was not detectable until 48 h of incubation in yeast extract sucrose (YES) agar, indicating reliance on prior *aflR* expression [125]. Interestingly, both research groups used beta-tubulin as a reference gene [47,125], demonstrating that their experimental conditions might have had a profound effect on gene expression of similar target genes. However, of greater interest and concern is the apparent differences in beta-tubulin expression in Abdel-Hadi et al.’s paper, as indicated by the changes in band intensity in Figure 4 of their study [47]. It seems that the expression of the reference gene changed substantially from week 2 to 3 at 85_W_ [47], further suggesting that beta-tubulin expression stability was not validated by the authors, and that its expression was not stable under their experimental conditions. In this study’s conditions, beta-tubulin was unlikely to be an appropriate reference gene.

Similarly, using beta-tubulin as a reference for normalisation, Fattahi et al. found that there was a significant increase in *cyp51A* expression in natural voriconazole-resistant *A. flavus* isolates, following growth in liquid media at 30 °C [51]. Their results were consistent with studies examining mutations in *cyp51A*, contributing to voriconazole resistance in *A. fumigatus* [126] and *A. lentulus* [127], as well as those examining laboratory-induced resistance in *A. flavus* [128]. However, Liu et al. found that *cyp51A* did not contribute to voriconazole resistance in *A. flavus* BMU29791, a strain isolated from a patient with invasive aspergillosis [129]. In their study, Liu et al. treated *A. flavus* with 0.25 μg/mL of voriconazole in minimal media, and used actin as a reference gene, though they also did not provide experimental evidence for validation of its stability [129]. Since the reference genes were not experimentally demonstrated to be stable under the tested conditions in either case [51,129], instead of different mutations causing the drug resistances among strains, alternative explanations such as differences in stability of reference genes in the experimental conditions cannot be ruled out. If the beta-tubulin and actin genes were validated under the experimental conditions used in these studies, it would be easier to determine whether the differences observed were caused by genetic differences among the strains and species.

### 2.2. Actin

Actin forms microfilaments and is among the most abundant proteins in eukaryotic cells [130]. Actin was first used as a reference gene in gene expression studies as early as 1985 [131], and similar to beta-tubulin, actin was first used as a reference gene in RT-qPCR studies as early as 2000 [132].

In our literature search, we found 17 reported usages of actin [16,21,23,25,26,34,42,43,60,75,77,78,79,89,91,96,108], 12 usages of beta-actin [22,24,44,45,61,76,90,97,103,104,105,107] and one reported usage of gamma actin [30] as reference genes. Unlike in the human genome, which contains about 20 copies of actin genes that make up six different isoforms of actin proteins [133], most fungi are known to contain only one actin gene [134,135]. Thus, we were curious as to how similar the actin and beta-actin genes were in *Aspergillus* fungi. We first checked the specificity of the primers used in these studies using Primer-Blast [136], and found that several of the primer sequences listed for beta-actin matched an actin gene instead, and one study matched both actin and beta-actin genes. We then conducted a BLASTn of the actin gene (Afu6g04740) of *A. fumigatus* Af293 in FungiDB. The sequence matched the gamma-actin gene (P168DRAFT_280232) in *A. campestris* with 87% identity, the actin gene (ACLA_095800) in *A. clavatus* with 94% identity and the actin gene (NFIA_051290) in *A. fischeri* with 99% identity. The sequence also matched the gamma actin gene, ANID_06542, for *A. nidulans* with 92% identity. The gamma actin gene in *A. nidulans* is the only actin gene in the fungus [135], and we will therefore include this reference gene with the counts for the actin reference gene in this review. We further ran a second BLASTn using the beta-actin sequence (accession number: AF276240) that was previously retrieved for *A. terreus* through Primer-Blast [136] (primers from Sorrentino and colleagues [107]), and compared the results to the BLASTn results for Afu6g04740. Similarly, the beta-actin sequence matched the gamma actin gene (P168DRAFT_280232) in *A. campestris* with 87% identity, the actin gene (ACLA_095800) in *A. clavatus* with 94% identity and the actin gene (NFIA_051290) in *A. fischeri* with 99% identity. Although the results from the FungiDB BLASTn did not contain an exhaustive list of all *Aspergillus* species, given that the two sequences BLASTed yield the same results in these three species, this demonstrates that actin and beta-actin are most likely the same gene. Here we follow the naming convention of *A. fumigatus* Af293, and call the gene actin. We have made this change in Table 1, reporting both actin- and beta-actin-encoding genes as actin (Table 1). We have kept the original gene symbols in Table S1 for the reader’s reference (Table S1).

Thus in total, actin was used in 30 studies examining gene expression in *A. niger* (3) [89,90,91], *A. nidulans* (4) [30,76,77,78,79], *A. fumigatus* (6) [16,21,22,23,60,61], *A. flavus* (4) [42,43,44,45], *A. parasiticus* (3) [103,104,105], *A. cristatus* (1) [24], *A. oryzae* (3) [25,96,97], *A. luchuensis* (2) [26,75], *A. terreus* (2) [107,108] and *A. aculeatus* (1) [34] (Table 1). Of these 30 studies, seven studies provided an explanation for using actin under the conditions tested [16,45,75,78,89,103,105], although only six of these studies provided experimental validation of its expression stability under the specific conditions [16,30,77,103,105,107] (Table 1, Table S1). Like those using beta-tubulin as a reference, many studies used RT-qPCR to investigate aflatoxin biosynthesis [42,44,45,103,104,105]. A few studies also focused on characterising putative biosynthesis pathway genes, such as those involved in trehalose synthesis [89], citric acid production [90] and galactofuranosylation [91], as well as the function of homologous genes in different species of *Aspergillus* [43,75]. Others looked to identify additional pathways contributing to antifungal resistance and potential antifungal drug targets [21,60,78,79], or tested the efficacy of natural antifungals [23,61]. Below in Section 2.2.1, we briefly describe the specific studies and experimental conditions that validated actin gene as an appropriate reference gene. Studies that did not provide validations are summarised in Section 2.2.2.

#### 2.2.1. Studies Validating Actin Expression Stability under the Experimental Conditions Tested

Of the 30 studies using actin as a reference gene, only six studies verified its stability under the experimental conditions tested [16,30,77,103,105,107] (Table 1, Table S1). Miao et al. followed the “gold standard” for reference gene selection by verifying the expression stability of two reference genes, actin, and the histone-encoding gene of *A. fumigatus* Z5 under the experimental conditions used in their study [16]. They used geNorm [33] to validate the two reference genes [16]. As such, actin has been demonstrated to be stably expressed during *A. fumigatus* Z5 growth in Mandels’ salt solution, supplemented with 1% oat spelts xylan, as the xylanase inducer [16]. Using RT-qPCR, Miao and colleagues found that 11 of the 13 xylanase-encoding genes in *A. fumigatus* Z5 are expressed in response to xylan, in addition to eight xylan-induced secreted proteins [16]. They identified four endoxylanases, two xylosidases, one α-L-arabinofuranosidase and one acetyl xylan esterase as important xylan-degraders, and thus promising agents for future biofuel generation [16].

Gao et al. also followed the “gold standard” for reference gene selection by validating the stability of actin in *A. nidulans* during its growth in the two media conditions, yeast extract-agar-glucose (YAG) with and without 15% (*w/v*) polyethylene glycol (PEG), employed in their study [77]. RT-qPCR analysis revealed that the expression of four members of the calcium signalling pathway—*midA*, *crzA*, *pkaA* and *pmrA*—significantly increased under osmotic stress in the presence of 15% PEG in *mobB*/*cotA* mutants of *A. nidulans* [77].

The gold standard was also applied by Deloménie et al., who also validated the expression stability of two reference genes, actin and GAPDH [30], using GeNorm [33]. They then used these genes to normalise gene expression in their custom Agilent microarray for *A. nidulans* [30]. They used RT-qPCR to verify the accuracy of the microarray data, by checking the expression of five genes: two that were stably expressed according to microarray analysis, ANID_08764 and ANID_05831; and three that increased in expression, ANID_00858, v00296, and ANID_02343 [30]. RT-qPCR analysis confirmed that ANID_08764 and ANID_05831 were stably expressed, and that ANID_00858, v00296 and ANID_02343 increased in expression, although the fold change was greater for RT-qPCR results [30]. As articulated by the researchers [30], this increase in fold change can be explained by the compression effect often observed for microarrays, and more severely in Agilent microarrays, when comparing RT-qPCR data [137]. The compression effect is attributed to technical limitations of microarrays, such as the limited dynamic range of signal intensities and cross-hybridisation among paralogous sequences [137].

Similarly, Sorrentino and colleagues validated the use of actin as a reference gene under the conditions used in their work, examining the addition of linoleic acid to enhance lovastatin production in *A. terreus* [107]. *A. terreus* is the main producer of lovastatin, a drug used to lower cholesterol [138]. Using RT-qPCR, Sorrentino and colleagues found that addition of linoleic acid increased the expression of two lovastatin biosynthetic genes, *lovB* and *lovF* [139], when compared to control cultures, while noting that the expression of actin remained consistent [107]. However, the group did not provide the data demonstrating stable actin expression [107]. Thus, fatty acids such as linoleic acid enhance lovastatin production, and this knowledge can be applied to industrial fungal fermentation as a cost-effective method for increasing lovastatin yield [107].

Ghanbari et al. also validated the stability of actin expression in their study, examining the efficacy of *Kluyveromyces lactis* as a biocontrol agent against aflatoxin production and *aflR* expression in *A. parasiticus* [105]. Consequently, actin expression has been shown to be stable during co-incubation with 1.5 × 10^5^ CFU/mL of *K. lactis* at 30 °C for 48 h [105]. They found that there was a significant decrease in *aflR* expression in cells treated with *K. lactis* compared to untreated cells, and that this corresponded with a decrease in aflatoxin production [105]. This indicates that *K. lactis* may decrease aflatoxin production through the downregulation of *aflR* [105].

Finally, Kondo et al. used actin as a reference gene to examine the effects of aflastatin A on aflatoxin expression in *A. parasiticus* [103]. The authors cite their previous work with the same species during exposure to blasticidin and its derivatives as justification for using actin as a reference gene [140]. Since the structures of aflastatin A and blasticidin are similar [141], hypothesising that actin expression would exhibit the same stability when exposed to alfastatin A is reasonable, and therefore the authors provide good justification for its use in their study. The group found that the expression of the aflatoxin biosynthesis genes *aflC*, *aflM*, *aflP* and *aflR* was significantly diminished following treatment with aflastatin A [103]. Moreover, they found that aflastatin A also decreased the expression of *aldA* and *facA* [103], which encode aldehyde dehydrogenase [142] and acetyl-CoA synthetase [143], respectively.

#### 2.2.2. Studies Missing Proper Actin Expression Stability Validation

Twenty-four of the 30 studies did not include proper expression stability validation of actin as a reference gene [21,22,23,24,25,26,34,42,43,44,45,60,61,75,76,78,79,89,90,91,96,97,104,108]. Trevisan et al. and Verheecke et al., who used RT-qPCR to examine the expression of genes involved in nutrient-sensing [76] and aflatoxin biosynthesis [42] (as discussed above), respectively, also used actin as an unvalidated reference gene. As with beta-tubulin, several studies investigated the expression of genes facilitating antifungal resistance [60,61,79] and aflatoxin biosynthesis [42,44,104]. Additionally, two studies used RT-qPCR to confirm RNA-sequencing (RNA-seq) data, including the expression of the growth phase-associated [21] and amylolytic enzyme [26] genes. Similarly, several studies used RT-qPCR to confirm microarray expression data, including the expression of members of the gliotoxin gene cluster [22], non-ribosomal peptide synthetases [23], sporulation-related genes [24] and highly expressed mating-type associated genes [25]. Others studied the expression of genes associated with G*alf* biosynthesis [78], nitrogen regulation and catabolism [43], citric acid production [75,90], spore color development [96] and conidiation [43,97], as well as putative trehalose synthesis genes [89] and one gene encoding alpha-glucan synthase [91]. Additionally, one study used RT-qPCR to verify increased transcription of genes associated with the industrially relevant product, taxol [108], while another study used RT-qPCR to characterise the *acr* biosynthetic cluster of a novel protein, Acurin A, in *A. aculeatus* [34].

As mentioned previously, failure to properly validate reference genes can have negative consequences on the interpretation of results. Bruns et al. used actin as a reference gene in their RT-qPCR experiment, aimed at verifying the expression of four gliotoxin cluster genes obtained using microarray [22]. However, the group did not experimentally validate the expression of actin prior to conducting RT-qPCR [22]. Bruns et al. noted that there was a discrepancy in the expression levels of *gliN*, *gliP*, *gliG* and *gliT* when comparing the data obtained using microarray to that obtained using RT-qPCR [22]. They noted that the limited dynamic range of detection for microarrays and differences in probe design might have contributed to the differences observed in gene expressions between methods [22]. However, as RT-qPCR was used by the research group to verify the results obtained using microarray [22], it would have been prudent for the researchers to ensure that the reference gene used for RT-qPCR exhibited stable expression under the conditions being tested.

Similarly, Perrin et al. used RT-qPCR to confirm the microarray expression data for seven non-ribosomal peptide synthetases and an additional four non-ribosomal peptide synthetases associated with siderophore biosynthesis [23]. They also used actin as a reference gene, but did not validate its expression stability under the conditions tested [23]. They report that the siderophore gene, *sidE*, reaches maximum expression during growth under low iron availability [23], conflicting with Reiber et al.’s finding that *sideE* is insensitive to changes in iron concentrations and is constitutively expressed, except under iron replete conditions (300 μM) at 24 h of growth [144]. Interestingly, Reiber et al. used calmodulin as the reference gene in their study, and demonstrated that calmodulin is constantly expressed in *A. fumigatus* under the conditions tested (mineral salt medium (pH 6.8), supplemented with 0, 20 and 300 μM) [144]. Curiously, Perrin et al. note that the differences in expression patterns observed between their study and Reiber’s group are likely due to subtle differences in the growth conditions, yet the media composition and iron concentrations used were the same in both studies. The only difference between the two studies was the shaking speed used, which was 230 rpm for Perrin et al. [23] and 280 rpm for Reiber et al. [144]. Therefore, the differences in observed *sidE* expression may be due to differences in reference gene expression, where actin expression differed under the experimental conditions used by Perrin et al. [23]. In fact, Perrin et al. note that actin expression decreased in the *laeA* mutant examined during growth under low iron availability, indicating that actin was likely not an appropriate reference gene for their study [23]. To ascertain the cause for the observed differential expression, the actin reference gene must be validated under the experimental conditions used by Perrin’s group. This case clearly illustrates the importance of reference gene validation in RT-qPCR analyses under the specific conditions being studied, and the consequences associated with improper reference gene use.

### 2.3. 18S rRNA

The 18S rRNA gene encodes the small ribosomal RNA subunit of the translation apparatus in all eukaryotes. The rDNA copy number of the 18S rRNA gene in *A. fumigatus* is strain-dependent, ranging from 38 copies in the Af293 strain to up to 91 copies in select strains [145]. Unlike single-copy genes, this variable nature in copy number among strains represents a potential drawback of using 18S rRNA as a reference in comparative analyses among strains. However, 18S rRNA is a highly conserved gene at the DNA sequence level, and has been used as a reference gene in gene expression analyses as early as 1990 [146]. Similar to the two previously discussed reference genes, beta-tubulin and actin, the 18S rRNA gene was first used in RT-qPCR analyses as early as 2000 [147]. Additionally, the product of the 18S rRNA gene is commonly used to confirm the overall integrity of the isolated RNA used in RT-qPCR [63,66].

In this review, we found that the 18S rRNA gene was used as a reference gene 12 times in studies examining gene expression in *A. niger* (2) [87,88], *A. fumigatus* (3) [27,58,59], *A. flavus* (4) [28,39,40,41], *A. parasiticus* (2) [29,102], *A. sojae* (1) [102], *A. oryzae* (1) [88] and *A. carbonarius* (1) [36] (Table 1, Table S1). Only two of the twelve studies validated the use of the 18S rRNA gene under the conditions being tested [27,41], with the remaining ten studies offering no explanation for its use as a reference gene [28,29,36,39,40,58,59,87,88,102] (Table 1, Table S1). As discussed above, several of these studies examined the efficacy of potential antifungals [27] and aflatoxin biosynthesis antagonists [40,41], as well as the influence of abiotic factors, such as temperature and water activity [28], on gene expression. Genetic alterations were also examined, including deletion mutants, to assess the importance of genes involved in aflatoxin biosynthesis [29], conidiation [29] and production of extracellular proteins [39], as well as overexpression strains, to assess the role of cofilin during oxidative stress and pathogenesis [59]. Two groups also examined the influence of carbon and nitrogen sources on the expression of genes in two relevant, yet different pathways: those involved in production of industrially useful xylanases [87]; and harmful allergen-encoding genes [58].

#### 2.3.1. Studies Validating 18S rRNA Expression Stability under the Experimental Conditions Tested

The 18S rRNA gene was only validated in two of the 12 studies examined in this review [27,41]. As mentioned in Section 2.1.1, Lappa et al. employed the “gold standard” for reference gene selection by validating the use of the three reference genes (beta-tubulin, 18S rRNA and calmodulin) under the experimental conditions used in their study examining aflatoxin biosynthesis [41]. Gautam et al. also used the “gold standard” for reference gene selection, by testing the stability of three candidate reference genes (actin, GAPDH and 18S rRNA) in preliminary experiments for their study investigating the efficacy of the antimalarial drug artemisinin against *A. fumigatus* [27]. Of the three candidate reference genes tested, the 18S rRNA gene exhibited the greatest stability [27]. Using RT-qPCR, seven of 745 genes displaying an altered expression following exposure to 125 μg/mL artemisinin, including the oxidative phosphorylation pathway-specific 64 kDa mitochondrial NADH dehydrogenase, were confirmed to exhibit a similar change in expression [27]. Interestingly, while the 64 kDa mitochondrial NADH dehydrogenase was downregulated upon artemisinin exposure, other oxidative phosphorylation genes were upregulated [27]. One explanation for this is that the 64 kDa mitochondrial NADH dehydrogenase is a specific target of artemisinin, and due to its downregulation, *A. fumigatus* overexpresses additional oxidative phosphorylation genes to equilibrate the membrane potential of the fungus [27]. Additionally, co-incubation with artemisinin and itraconazole produced a synergistic effect, suggesting that artemisinin may be useful in combination infection treatments with other azole antifungals [27].

#### 2.3.2. Studies Missing Proper 18S rRNA Expression Stability Validation

Ten of the twelve studies that used the 18S rRNA reference gene did not validate its stability under the experimental conditions of their study [28,29,36,39,40,58,59,87,88,102]. Like studies described above in Section 2.2, Chang et al. used RT-qPCR to confirm microarray expression data for genes associated with oxidative stress [29]. Similarly, the expression of aflatoxin biosynthesis genes was also examined in studies using the 18S rRNA gene as a reference [40,102]. These studies also included those investigating the expression of miRNA-like genes [28] and xylanases [87], as well as the expression of genes associated with allergies [58], oxidative stress and cell wall polysaccharide biosynthesis [59]. Other studies looked at the expression of differentially secreted proteins, such as alpha amylase, in response to the deletion of VeA [39] and putative ochratoxin A (OTA) biosynthesis genes [36]. In addition, one study evaluated the expression of single-guide RNA for use in CRISPR-Cas9 editing of *A. niger* [88].

### 2.4. GAPDH

Glyceraldehyde-3-phosphate dehydrogenase (*GAPDH*, also called *gpdA* and *gpdh*) plays a vital role in glycolysis by catalysing the conversion of glyceraldehyde-3-phosphate (G3P) to 1,3-biphosphoglycerate in the presence of NAD^+^ and inorganic phosphate [148]. The role of GAPDH extends to additional cellular processes, acting as a potential transcription factor (as demonstrated by binding to RNA polymerase II in *Schizosaccharomyces pombe*) [149], glucose availability sensor and corresponding regulator of cell growth [150], as well as apoptosis inducer [151].

GAPDH has been used in gene expression analyses as early as 1991 [152]. It was first used as a reference gene in RT-qPCR analyses as early as 1992 [153], making it the earliest reference gene used of four reference genes discussed in detail in this review. GAPDH was used as a reference gene in ten studies examining gene expression in *A. niger* (2) [90,92], *A. nidulans* (1) [30], *A. fumigatus* (3) [31,60,71], *A. flavus* (2) [52,54], *A. westerdijkiae* (1) [110] and *A. aculeatus* (1) [35] (Table 1, Table S1). These studies included those that investigated the function of proteins, including the type III polyketide synthase [92] and alternative oxidase [90] genes, using deletion and overexpression mutants. Several studies also used RT-qPCR to confirm the results from other gene expression analyses, including microarrays [30,31] and cDNA RDA [110]. Another study by Blosser and Cramer (also discussed above) examined the mechanism of triazole resistance in *A. fumigatus* mediated by SbrA [60]. Only four of the ten studies using GAPDH as a reference gene experimentally validated its stability [30,35,52,54] (Table 1, Table S1).

#### 2.4.1. Studies That Validated GAPDH Expression Stability under the Experimental Conditions Tested

Of the ten studies that used GAPDH as a reference gene, four studies experimentally validated its expression stability under the conditions being tested [30,35,52,54]. As mentioned above, in Section 2.1.1, Caceres et al. employed the “gold standard” for selecting reference genes when selecting beta-tubulin and GAPDH in their 2016 study, examining the effects of 0.5 mM eugenol on expression of aflatoxin biosynthesis genes [52]. These same reference genes were also used in their latter study examining the impact of *S. roseoulus* on aflatoxin biosynthesis gene expression [54]. Similarly, Deloménie et al., also mentioned above in Section 2.1.1, employed the “gold standard” for reference gene selection by first validating the expression stability of actin and GAPDH [30].

Tani et al. also validated the expression stability of GAPDH as a reference gene using NormFinder [119], in their study investigating the role of the XlnR signalling pathway in *A. aculeatus* [35]. Through examining the expression of cellulase and hemicellulase genes (*bgl1*, *cbhI*, *cmc1*, *cmc2*, *xynIa*, and *xynIb*) in *A. aculeatus* wildtype and Δ*xlnr* strains, during growth in the presence of various carbon sources (1% (*w/v*) polypeptone, 1% (*w/v*) glucose, 1% (*w/v*) avicel, 1% (*w/v*) xylose, or 1% (*w/v*) arabinose), the researchers demonstrated that there are two different pathways mediating cellulase and hemicellulase expression [35]. Specifically, there is one pathway that mediates the expression of *cbhI*, *cmc2* and *xynIa* in minimal media containing 1% (*w/v*) avicel, which is independent of XlnR signalling [35]. Moreover, expression of *cbhI*, *cmc2* and *xynIa* was induced by 30 mM cellobiose in the wildtype strain, whereas *xynIb* and *cmc1* expression was not, suggesting that cellobiose is an exclusive inducer of the XlnR-independent pathway [35]. This study was the first to report diverging routes of gene induction via cellulose, to XlnR-dependent and -independent pathways, and makes a valuable contribution to our understanding of transcriptional inducers in filamentous fungi [35].

#### 2.4.2. Studies Missing Proper GAPDH Expression Stability Validation

The remaining six studies using GAPDH as a reference gene did not validate its stability under the conditions tested [31,60,71,90,92,110]. Blosser and Cramer and Hou et al., who investigated the expression of genes involved in antifungal resistance [60] and citric acid production [90] using RT-qPCR and actin, as an unvalidated reference gene, respectively, also used GAPDH as a reference gene. Blosser and Cramer also used a third reference gene in their study, *tefA*, although this gene was not experimentally validated either [60]. As with the sections above, the expression of genes contributing to antifungal resistance [60,71] was examined. Again, RT-qPCR was used to verify the results of other methods of gene expression analysis, including the change in expression in eight genes in *A. fumigatus* conidia exposed to human airway epithelial cells [31] and three putative oxidoreductases in *A. westerdijkiae* [110]. Additionally, one study investigated the expression of the type III polyketide synthase, *AnPKSIII* [92].

Oosthuizen et al. investigated dual organism transcriptomics, looking at the transcriptome of *A. fumigatus* conidia and *A. fumigatus*-infected human airway epithelial cells (AECs) using microarrays [31]. They used RT-qPCR to examine a representative group of genes for both *A. fumigatus* conidia incubated with AECs or human bronchial epithelial cells (16HBE14o-) and a representative group of genes expressed in AECs and 16HBE14o-[31]. For analysis of *A. fumigatus* conidia, this representative group of eight genes included those involved in vacuolar acidification and those that possess metallopeptidase activity [31]. Three of the eight *A. fumigatus* genes—vacuolar ATPase 98 kDA subunit, *MAP-1* and *SkpA*—were significantly upregulated following incubation with either AECs or 16HBE14o- [31]. In contrast, tubulin-specific chaperone C and β-glucosidase significantly increased in expression, but only when incubated with AECs [31]. Similarly, *SidA* and *fdh* significantly increased in expression when incubated with 16HBE14o- cells [31]. Overall, microarray and RT-qPCR analysis elucidated similar trends in expression [31]. Through looking at the expression of genes in human epithelial cells and *A. fumigatus* conidia simultaneously, the researchers provide a better representation of the changing expression dynamics as host and pathogen interact [31]. 

Hou et al. characterised the role of the mitochondrial alternative oxidase gene, *aox1*, in citric acid production in *A. niger* [90]. Interestingly, the researchers did not validate the expression stability of either of their two reference genes, actin and GAPDH, under the experimental conditions used [90]. They compared the expression of six metabolic genes—*aox1*, *cox*, *cat*, *hk*, *pfk* and *cs*—in an *aox1* overexpression strain (102) and an Δ*aox1* strain (3–4), to each other, as well as the parental strain, CGMCC 10142 [90]. In strain 3–4, *cox* expression increased compared to the parental strain, with a coinciding increase in ATP concentration for all sampled time points, except for 36 h and 48 h [90]. In strain 102, the expression of *cox* decreased compared to the parental strain [90]. In the absence of *aox1*, *cat* expression was more constant and relatively higher during aerobic treatment, in relation to the parental strain [90]. Correspondingly, a negative correlation between *aox1* and *cat* expression was observed in strain 102, compared to the parental strain [90]. The expression of *hk, pfk* and *cs* genes was higher in strain 102 compared to strain 3–4 [90]. Citric acid was also produced at higher quantities in strain 102 [90]. This work establishes the linkage between *aox1* expression, genes involved in citric acid production, and the amount of citric acid produced, using methodology that can be applied to other industrially relevant biological mechanisms [90].

### 2.5. Others

Other reference genes that have been used over the last two decades to study gene expression in *Aspergillus* include those that encode: histones [16,34,38,82,93,94,99,100,101]; nicotinamide adenine dinucleotide phosphate^+^ hydrogenase [85]; translation elongation factors [84] and elongation factor subunits [60,70,73,74]; ribosomal proteins [86]; putative 1,3-beta-glucan synthase catalytic subunit [72]; calmodulin [37,41,53,95]; internal transcribed spacer regions [57]; and ubiquitin-conjugating enzyme [32] (Table 1, Table S1).

Of those using the above-mentioned reference genes, four studies used multiple reference genes to normalise their expression data [16,41,60,82]. Six different studies experimentally validated the expression stability of the reference genes used under the conditions tested [16,32,38,41,86,94]. Several of the studies have already been discussed above [16,38,41], and we will therefore focus here on three studies that validated the expression of the reference genes used in their work identifying and characterising the conserved *WDR* gene (*FPWDR*) in *A. nidulans* [86], validating RNA-seq data examining OTA biosynthesis in *A. carbonarius* [32] and modelling the expression dynamics of the XlnR regulon in *A. niger* [94].

Prior to assessing the impact of deleting *FPWDR* and the surrounding locus, on the expression of cell wall-associated genes in *A. nidulans,* Guerriero et al. used geNormPlus [33] to rank the expression stability of five candidate reference genes—*rpl37*, *rpl3*, actin, *CRP2* and *TEF1*—during growth in standard minimal media [86]. As *rpl37* and *rpl3* were the most stable, they were both chosen as reference genes for normalisation [86]. Following genomic analysis of *FPDWR*, Guerriero et al. deleted *FPWDR* and the surrounding locus (including *bf*), generating a heterokaryon transformant (hkΔAN1556) [86]. Since hkΔAN1556 possessed deformities in cell wall morphology [86], the group decided to look at the effect of the deletion on the expression of cell wall-associated genes, including members of the *chs* family, *csmA*, *csmB*, *celA*, *fksA*, *rhoA* [154], *pkcA* [155], *wscA*, *wscB* [156], *CPS1* and *bf*. Generally, the expression of cell wall-associated genes decreased in hkΔAN1556, with a significant reduction in *chsED*, *csmA*, *csmB*, *CPS1*, *fksA* and *wscB* expression [86]. Surprisingly, *FPWDR* and *bf* expression was similar in hkΔAN1556 compared to the control strain, which suggests that there are nuclei within hkΔAN1556 where *FPWDR* and the surrounding locus were not deleted, and thus their expression is retained [86]. Taken together with its proximity to the *chsD* gene and the presence of cell wall deformities, the alterations in gene expression observed for hkΔAN1556 indicate that *FPWDR* plays a role in the cell wall [86].

Gerin et al. used RT-qPCR to confirm their RNA-seq data, demonstrating differential expression of five polyketide synthases, four non-ribosomal peptide synthetases and one chloroperoxidase in *A. carbonarius* during OTA-inducing conditions [32]. To determine the best reference gene for normalisation, the group assessed the expression stability of beta-tubulin, calmodulin and ubiquitin-conjugating enzyme during *A. carbonarius* growth in minimal media at 25 °C in the dark without shaking, using BestKeeper [157]. Of the three candidate reference genes tested, the ubiquitin-conjugating enzyme was the most stable, and therefore was used to normalise their RT-qPCR data [32]. The RT-qPCR analysis demonstrated similar patterns of gene expression as observed for RNA-seq, after *A. carbonarius* growth in minimal media in the dark at 25 °C without shaking for four, six and eight days [32]. Overall, their results provide a comprehensive view of the different expression networks that may be connected to OTA production [32].

Omony et al. used the histone-encoding gene *hist* in their work building off their previous studies [158,159], and modelling the transcription dynamics of 23 genes in the XlnR regulon using time-course RT-qPCR expression data of *A. niger* [94]. The group previously experimentally validated the expression of the histone-encoding gene under the same experimental conditions, treatment with 1 mM or 50 mM D-xylose, and in the same strains used in their study, *A. niger* N400 (wildtype) and NW28 (a derepressed *CreA* mutant) [158]. As in their study four years earlier [158], Omony et al. found that the expression of XlnR regulon was higher for the 1 mM D-xylose treatment in both the wildtype and mutant strains [94]. They also found that the induction of hemicellulose genes was higher for the 1 mM D-xylose condition, and that *xlnR* expression was constant, regardless of functional *CreA* or D-xylose concentration [94]. The group also presents an updated kinetic differential equation model for the transcription of the XlnR regulon [94]. As the general results of their two studies were consistent, this is excellent support for the use of the histone-encoding gene for examining XlnR regulon transcription in *A. niger*, using 1 mM and 50 mM D-xylose for transcriptional induction [94,158].

## 3. Validation of Candidate Reference Genes in *Aspergillus*

The studies highlighted above use RT-qPCR to answer a diversity of research questions about gene expression in various species of *Aspergillus* under specific experimental conditions. In this section, we will highlight the need for studies validating the expression stability of candidate reference genes under standard laboratory conditions by discussing two large-scale validation studies in *A. flavus* and *A. niger*. We will also summarise all reference genes experimentally validated to date with their associated species and experimental conditions, and highlight the traditional reference genes that were shown to be less stably expressed than the selected reference gene under the specific tested experimental conditions.

### 3.1. Validation of hisH4 and cox5 for Studying Aflatoxin Biosynthesis in A. flavus

From our PubMed search, only one study was returned that assessed the expression of multiple candidate reference genes under a specific set of conditions [111]. To find reference genes suitable for RT-qPCR analysis of aflatoxin biosynthesis genes in *A. flavus*, Suleman and Somai tested the expression stability of four reference genes (*actA* (actin), *sarA* (secretion associated binding protein), *hisH4* (histone H4) and *cox5* (cytochrome C oxidase subunit V)) under aflatoxin-inducing, growth in sucrose low salts (SLS) or sucrose low salts supplemented with 117 mM ammonium sulphate (SLS + NH_4_), as well as non-inducing growth in lactose low salts (LLS), conditions at acidic (pH = 4.0) or alkaline (pH = 8.5) pH [111]. They chose actin, *sarA* and *cox5* for testing, as their expression stability was previously demonstrated in *A. niger* [160], and *hisH4*, as it had been previously used to normalise expression in *A. oryzae* [161].

Prior to assessing the stability of the candidate reference genes, Suleman and Somai assessed the primer pairs for each gene [111]. An ideal primer pair should: (1) produce an amplification efficiency of ~100% (indicating a doubling of product per cycle); (2) produce a standard curve with a slope of −3.32, y-intercept less than 40 and correlation coefficient of >0.990; and (3) amplify one product (as indicated by a single peak in melt-curve analysis) [19,162]. The amplification efficiencies of *cox5* and *hisH4* were ~100%, while the amplification efficiencies of *sarA* and actin were less than 80%, despite attempts to redesign and optimise the primers [111]. Therefore, only *cox5* and *hisH4* were assessed further [111]. As the standard deviations of the Cq values was less than one for both *cox5* and *hisH4* when comparing different treatment conditions, BestKeeper and REST2009 analyses demonstrated that there was no significant change in expression for either gene [111].

As mentioned in the introduction above, using multiple, validated reference genes is the MIQE recommended practice for normalising gene expression data [19]. Thus, to assess whether normalisation with one reference gene (*cox5* or *hisH4*) or both reference genes together yielded more robust results, the researchers normalised the expression of a “dummy” reference gene, with a set Cq value of 15, to the experimentally obtained Cq values of each reference gene, as well as to the sum of their Cq values, using REST2009 [111]. Using this method, the “dummy” gene should not exhibit a change in expression (expression ratio of ~1.0 with a *p*-value > 0.055) [163]. When comparing normalisation with *hisH4* or *cox5* alone, to normalisation with both *cox5* and *hisH4* together, a better overall expression ratio was observed when using both reference genes for normalising the data from acidic and alkaline conditions [111]. When assessing the expression data under specific conditions, they found that normalisation with both reference genes also produced the best expression ratio for SLS + NH_4_ [111]. However, under three sets of conditions (when normalising expression data following growth on SLS under acidic and alkaline conditions; when comparing expression during growth on SLS + NH_4_ to SLS under acidic and alkaline conditions; and when comparing expression during growth on LLS to SLS under acidic conditions), normalisation with *hisH4* yielded the best expression ratio [111]. Normalisation with *cox5* only yielded a better expression ratio for data obtained following growth on LLS, as well as when comparing expression during growth on LLS to SLS under alkaline conditions [111]. Collectively, these results show that the use of multiple reference genes is not always optimal for the conditions being tested [111]. Thus, this study highlights the need to validate the stability and the utility of reference genes under the specific conditions of each experiment.

### 3.2. Validation of actA, sarA and cox5 for Studying glaA Expression in A. niger

Bohle et al. experimentally validated the expression stability of ten candidate reference genes (*actA*, *sarA*, *cox5*, *apsC* (aminopeptidase C), *gpd* (GAPDH), *glkA* (glucokinase), *g6pdh* (glucose-6-phosphate dehydrogenase), *icdA* (isocitrate dehydrogenase precursor), *pfkA* (phosphofructokinase) and *pgiA* (phosphoglucose isomerase) in *A. niger* during growth in batch and continuous cultures, with *glaA*-inducing and -non-inducing conditions [160]. They first examined the expression stability of each gene in fedbatch cultures of *A. niger*, with glucose as the carbon source and continuous glucoamylase (*glaA*)-induction, for nine time points using geNorm [160]. Interestingly, one of the most used reference genes, *GAPDH* (used in ten studies highlighted in this review [30,31,35,52,54,60,71,90,92,110], two of which examined expression in *A. niger* [90,92] specifically) was the least stable under these conditions [160]. This further illustrates the importance of experimentally validating commonly used reference genes for use in study-specific conditions.

Since the researchers were interested in examining expression stability for a diverse range of experimental conditions, they decided to examine the six most stably expressed genes, *act*/*sarA*, *g6pdh*, *cox5*, *apsC* and *pgiA* (most to least stable), under *glaA*-inducing (glucose as the carbon source) and -non-inducing (xylose or maltose as the carbon source) conditions in modified Vogel-Medium with stir speeds, and pH varying from 400–1000 per min and 3.0 to 5.5, respectively [160]. They found *actA* and *sarA* to be the most stably expressed, followed by *cox5* [160]. Since their dataset contained more samples for *glaA*-inducing conditions, they repeated their analysis with an equally represented dataset to ensure that the same three genes were consistently the most stable, irrespective of induction [160]. They found that the order of stability differed following the second analysis, where *act* and *cox5* exhibited the greatest stability, followed by *sarA* again, demonstrating the influence of experimental conditions on reference gene stability [160].

To further investigate the potential advantage of normalisation using three validated reference genes, they compared the correlation coefficients computed following regression with the normalisation factor for *act*, *sarA* and *cox5* (N*_act_*_,*sarA*,*cox5*_), the unvalidated reference gene, GAPDH (N_GAPDH_) and total RNA [160]. N*_act_*_,*sarA*,*cox5*_ resulted in the highest correlation coefficient, demonstrating that the combination of the three validated reference genes was the best approach under the experimental conditions [160].

An important consideration noted by the authors is that these genes are all from different functional classes, and thus co-regulation of these genes is highly unlikely [160]. The absence of co-regulation is critical when using geNorm, as co-regulated genes can lead to high stability ranking and the inclusion of false positives [33]. Therefore, this work presents three experimentally validated reference genes that are suitable for studying *glaA* expression, while continuing to highlight the crucial first step of experimentally validating reference genes.

### 3.3. Reference Genes Currently Validated for Use in Aspergillus

Based on our review of the literature in Section 2, Section 3.1 and Section 3.2 above, the genes shown in Table 2 are recommended as reference genes for the specified experimental conditions. In this table, we also provide the species that these genes have been validated for, as well as the primers for each species and each recommended reference gene (Table 2). Table 2 is organised alphabetically by species first and reference gene second, followed by date. The information in Table 2 was extracted from 21 publications [16,27,30,32,35,38,41,46,52,54,55,77,86,94,103,105,107,111,141,144,160].

While the reference genes described above are validated for use under the specific conditions described in Table 2 below, three papers that tested the expression stability of multiple candidate reference genes demonstrated that several traditionally used reference genes were less stably expressed under the experimental conditions tested than the reference genes that they chose [27,32,86]. In their preliminary experiments, Gautam et al. found that actin and GAPDH were less optimal reference genes than the 18S rRNA gene for RT-qPCR gene expression analysis of *A. fumigatus* during exposure to 125 μg/mL artemisinin or solvent control for 3 h at 37 °C [27]. Similarly, Gerin et al. found that beta-tubulin and calmodulin were less suitable reference genes than the ubiquitin-conjugating enzyme for RT-qPCR gene expression analysis of *A. carbonarius* during growth in minimal media under OTA-inducing conditions for four, six and eight days in the dark at 25 °C [32]. Guerriero et al. also demonstrated that actin was less optimal for assessing gene expression in *A. nidulans* during growth in liquid minimal media under standard conditions, than two putative ribosomal proteins, L37 and L3 [86]. These two putative ribosomal genes were also found to be more suitable reference genes than *CRP2* and *TEF1* under these experimental conditions [86].

Additionally, as highlighted in the candidate reference gene validation study by Bohle et al. and discussed in Section 3.2 above, a traditionally used reference gene, *GAPDH*, was found to be the least stable candidate reference gene in feed-batch cultures of *A. niger*, with glucose and continuous *glaA*-induction [160]. Additionally, the candidate reference genes, *icdA*, *glkA* and *pfkA* (most to least stable), demonstrated low stability, and are therefore not the most suitable reference genes for use under these conditions [160]. Under *glA*-inducing and non-inducing conditions in modified Vogel-Medium with a pH range of 3.0–5.5 and stir speed of 400–1000 per minute, the candidate reference genes *pgiA*, *apsC* and *g6pdh* (most to least stable) were demonstrated to be less stable than actin, *sarA* and *cox5* [160].

## 4. Reference Gene-Specific Google Scholar Queries

**Figure 2:**
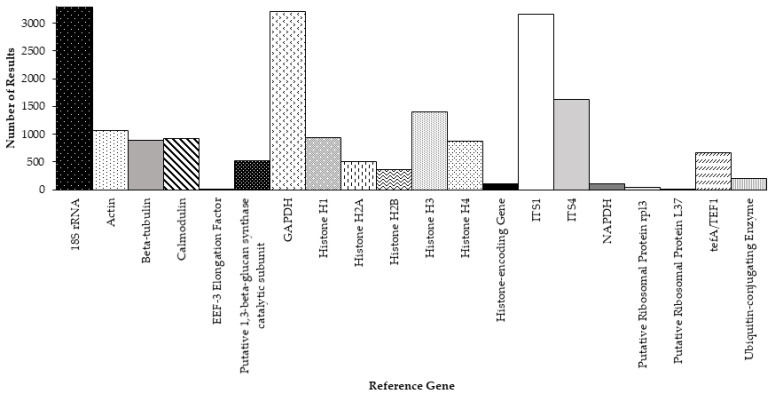
Number of search results for each reference gene based on their corresponding Google Scholar query. The 18S rRNA reference gene returned the most results, with 3290 results returned.

In this study, we focused our analyses on PubMed search results. However, though PubMed is a major database for the biomedical literature, there are other databases. As a broader search for RT-qPCR studies of *Aspergillus* fungi, we conducted an additional set of queries using Google Scholar, with the following structure: “*Aspergillus* “reference gene” qPCR”, where “reference gene” corresponds to the gene symbol associated with the reference gene of interest. We added quotation marks around the reference gene symbol to specifically search for the reference gene symbol as an entire string, and ensure that the substrings comprising the reference gene symbol would not be searched separately. For example, the 18S rRNA gene was queried as “*Aspergillus* “18S rRNA” qPCR” and not “*Aspergillus* 18S rRNA qPCR”. The dates of the returned results of the queries ranged from 1983 to 2021. Figure 2 summarised the search results for all reference genes reviewed in our initial PubMed searches. The Google Scholar query that returned the most results corresponded to the 18S rRNA gene, returning 3290 results total (Figure 2). This was followed by the query for GAPDH with 3209 results, *ITS1* with 3170 results and *ITS4* with 1630 results (Figure 2).

It is important to note that the search queries described in this section have several limitations. One limiation is that because qPCR is also used as a diagnostic tool for the detection and quantification of *Aspergillus* species [164], the results of each query likely contain these studies, as well as relevant gene expression studies. Additionally, any papers that contain “*Aspergillus*”in the body of their report, such as the introduction, but do not specifically examine gene expression in *Aspergillus*, may also be returned by the queries. Therefore, while the total results returned by each query may be used as a proxy for reference gene use frequency in qPCR gene expression analysis, the Google Scholar search results require manual curation as was done during the original PubMed search described in Section 2 above, in order to determine the relevance of the Google Scholar search results literature to our current study.

## 5. Concluding Remarks and Recommendations

In a recent 2018 review of reference gene validation practices for RT-qPCR of insects, Shakeel and colleagues discuss the validation of reference genes thus far for select insect species, while emphasising the need for a comprehensive group of studies to be conducted under diverse experimental conditions for all species of insects [165]. They note that several studies of classical housekeeping genes show varying expression under different experimental conditions, and indicate ribosomal genes as a promising new set of genes for further stability analysis in insect-specific studies [165].

In the literature reviewed above, we discussed several methods employed by those who validated the reference genes for normalisation in their studies, including GeNorm [33], BestKeeper [157] and NormFinder [119]. Shakeel and colleagues discussed the benefits of these and other methods, RefFinder and ΔCt, for assessing reference gene stability, and noted how each method may lead to slight differences in reference gene stability rankings [165]. As stated by the group, both GeNorm and Normfinder are excellent programs for the initial assessment of candidate reference gene stability, each with their own advantage, with GeNorm capable of determining the number of reference genes to use [33], and NormFinder computing the stability of each reference gene separately to avoid the consequences associated with co-regulation [119]. Given that different programs for determining reference gene stability may yield different stability rankings, we recommend that researchers use more than one program to validate the stability of the reference genes used under the experimental conditions being tested. In agreement with Shakeel and colleagues, because the results of some programs, such as GeNorm, may be biased due to co-regulation, care should be taken to select candidate reference genes that are not co-regulated.

Interestingly, Shakeel and colleagues noted nearly the same four reference genes as those most used in RT-qPCR studies in general across organisms: beta-actin, GAPDH, beta-tubulin and 18S rRNA, citing papers as early as 2004 [165]. Our examination of 90 RT-qPCR studies, spanning 2001 to 2020, further demonstrates this to be the case for *Aspergillus*. The authors note that as of 2000, beta-actin and GAPDH were used 90% of the time without proper validation [165]. Out of the 30 usages of actin and 10 usages of GAPDH across the 90 studies we examined, actin was used without validation approximately 83% of the time, and GAPDH 60%. Similarly, of the 31 usages of beta-tubulin and 12 usages of 18S rRNA across the 90 studies we examined, beta-tubulin was used without validation approximately 81% of the time, and 18S rRNA, approximately 83%.

**Figure 3:**
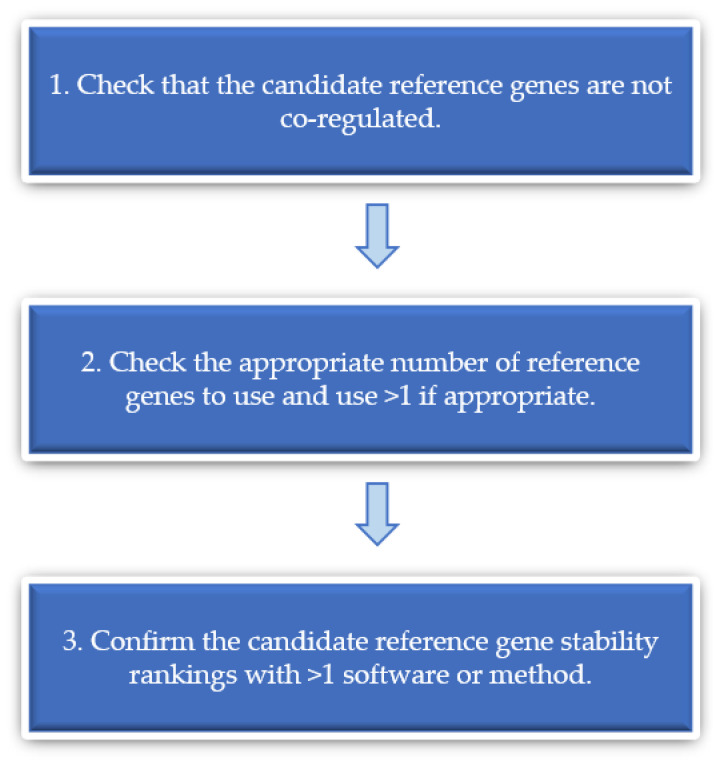
Recommended checkpoints to use when selecting reference genes.

Since its publication in 2018, this article by Shakeel and colleagues on insects has been cited 22 times by articles in PubMed. Fourteen of these citing articles are those evaluating candidate reference genes [166,167,168,169,170,171,172,173,174,175,176,177,178,179]. We hope that our critical review will similarly stimulate future research on experimentally validating reference genes for gene expression studies in *Aspergillus* (and in fungi in general) using RT-qPCR. Without experimental validation of reference genes, it can be difficult to interpret the potential contributors to expression differences among strains, genes and treatments. Figure 3 below summarises our recommended practice for reference gene selection.

## Figures and Tables

**Table 1:** Species, reference gene(s) and experimental conditions used in 90 RT-qPCR studies of *Aspergillus*.

Species	Reference Gene	Validation (Y/N)	Experimental Conditions	Ref.
*A. aculeatus*	Actin	N	Minimal media (MM) for 3, 5 and 7 days	[34]
	Histone 3			
	GAPDH	Y	MM with 1% (*w/v*) polypeptone, 1% (*w/v*) glucose, 1% (*w/v*) avicel, 1% (*w/v*) xylose, or 1% (*w/v*) arabinose for 3 h or 6 h	[35]
*A. carbonarius*	18S rRNA	N	Czapek-Dox Modified Yeast Agar (CYA) medium in the dark at 30 °C for 2 days	[36]
	Calmodulin	N	Co-culture with the actinobacterial strain, SN7, on International Streptomyces Project-2 (ISP2) media at 28 °C for 4 days	[37]
	Ubiquitin-conjugating Enzyme	Y	MM at 25 °C, without shaking (ochratoxin A (OTA)-inducing conditions) for 4, 6 and 8 days in the dark	[32]
*A. cristatus*	Actin	N	Cellulose membrane on malt yeast agar (MYA) and on 17% NaCl MYA media for 5 days at 28 °C in the dark, after which mycelia were fixed (extraction from 7-day mycelia)	[24]
*A. fischeri*	Beta-tubulin	Y	Growth on a hydrophobic polyvinylidene fluoride (PVDF) membrane on top of oatmeal agar for 3, 6 or 30 days (wildtype only)	[38]
	Histone 3			
*A. flavus*	18S rRNA	N	Glucose minimal media (GMM) containing 1% starch or 24 g/400 mL ground corn seed at 30 °C for 24, 48 and 72 h with shaking at 250 rpm	[39]
	18S rRNA	N	Yeast extract sucrose (YES) medium at 37 °C for 1.5 days and at 28 °C for 3 days, in the dark	[28]
	18S rRNA	N	YES media supplemented with 0.40 mmol/L of cinnamaldehyde, 0.56 mmol/L of citral, and 0.80 mmol/L of eugenol for 7 days	[40]
	18S rRNA	Y	Co-culture with *Listeria monocytogenes* in malt extract broth (MEB) at 25 °C and 30 °C for 7 days	[41]
	Beta-tubulin			
	Calmodulin			
	Actin	N	Co-incubation with soil isolates of *Streptomyces* on ISP2 medium at 28 °C	[42]
	Beta-tubulin			
	Actin	N	(1) Potato dextrose agar (PDA) or GMM supplemented with NH_4+_ for 48 h (2) GMM supplemented with 50 mM ammonium or NaNO_2_ for 30 h	[43]
	Actin	N	YES media containing 1.5 mg/100 mL silver nanoparticles at 28 °C for 14 days	[44]
	Beta-tubulin			
	Actin	N	YES or yeast peptone dextrose (YPD) media for 5 and 7 days, respectively, at 37 °C in the dark	[45]
	Beta-tubulin	Y	Inoculated onto 25 g of wheat and grown at 30 °C in open petri dishes with wetted filter paper for 9 days	[46]
	Beta-tubulin	N	Peanut samples for 6 weeks at 25 °C in polyethylene sandwich boxes containing glycerol/water solutions to maintain the equilibrium relative humidity conditions	[47]
	Beta-tubulin *	N	YES or yeast extract peptone (YEP) media at 28 °C for 4 days	[48]
	Beta-tubulin	N	Sucrose magnesium sulphate potassium nitrate yeast (SMKY) liquid media 25 °C for 7 days	[49]
	Beta-tubulin *	N	Treatment or no treatment with the subinhibitory concentrations of carvacrol or trans-cinnamaldehyde in potato dextrose broth (PDB) at 25 °C for 5 days	[50]
	Beta-tubulin	N	Liquid media at 30 °C with constant shaking at 120 rpm for 20 to 24 h	[51]
	Beta-tubulin	Y	Malt extract agar (MEA) media supplemented with 0.5 mM eugenol for 4 days at 27 °C in the dark	[52]
	GAPDH			
	Beta-tubulin	N	Coconut agar at 25 °C for 2 or 7 days	[53]
	Calmodulin			
	Beta-tubulin	Y	Co-incubation with *Streptomyces roseolus* on ISP2 medium at 30 °C for 4 days at a relative humidity of 80% in a Vötsch chamber	[54]
	GAPDH			
	Beta-tubulin *	Y	YES medium containing one of four antifungal peptides: PPD1, 66-10, 77-3, or D4E1	[55]
	Beta-tubulin	N	PDB containing 30% (*v*/*v*) of *Eurotium cristatum* culture filtrate for 3 days in the dark at 28 ± 2 °C with shaking at 120 r/min	[56]
	*ITS1*	N	Cultures of differing water potential were supplemented with different antioxidant concentrations at 20 °C and 28 °C for 72 h	[57]
	*ITS4*			
*A. fumigatus*	18S rRNA	Y	Treatment with 125 μg/mL artemisinin or solvent for 3 h in Roswell Park Memorial Institute (RPMI) 1640 medium at 37 °C	[27]
	18S rRNA	N	PDA with different carbon dioxide concentrations and on Czapek media with different C: N ratios for 48 h and 5 days	[58]
	18S rRNA	N	Liquid *Aspergillus* minimal media (AMM) at 37 °C for 18 h with shaking at 200 rpm	[59]
	Actin	N	(1) GMM at 25 °C for 60 days with shaking at 280 rpm (2) High iron liquid media or low iron liquid media at 37 °C for 24 h with shaking at 280 rpm	[23]
	Actin	N	Biofilm growth at 37 °C in minimal essential medium (MEM) supplemented with 5% *v*/*v* fetal calf serum (FCS) or 5% *v*/*v* phosphate-buffered saline (PBS) for 24 h and 48 h	[22]
	Actin	N	(1) CBS 144.89 and Δ*srbA*: Treatment with MIC_50_ of fluconazole or voriconazole in liquid GMM at 37 °C with shaking at 300 rpm CBS 144.89, Δ*srbA* and pniiA-erg11A-ΔsrbA: Liquid GMM plus 20 mM NO_3_ for 12 h and liquid GMM plus 20 mM NH_4_ at 37 °C with shaking at 300 rpm	[60]
	GAPDH			
	*tefA*			
	Actin	Y	Mandels’ salt solution with 1% oat spelts xylan for 0, 2, 4, 6 and 17 h	[16]
	Histone H4			
	Actin	N	RPMI medium at 37 °C for 0, 2, 4, 6, and 8 h	[21]
	Actin	N	Treatment with 24,700, 12,300 and 6170 µg/mL kombucha during growth in RPMI medium at 35 °C for 48 h	[61]
	Beta-tubulin	N	Yeast extract-peptone-dextrose (YEPD) media with 10 μg/mL (H11-20) or 100 μg/mL of itraconazole for 8 h at 37 °C	[62]
	Beta-tubulin	N	Treatment with 0.5 μg of voriconazole or without voriconazole in yeast glucose (YG) medium for 30, 60, 120, and 240 min	[63]
	Beta-tubulin	N	AMM containing 0, 1, 10, 100, 1000 μM of FeSO_4_ at 37 °C for 24 h with 150 rpm shaking	[64]
	Beta-tubulin	N	MEM supplemented with 10% (*v*/*v*) human serum (male) and 50 μM FeCl_3_ at 37 °C for 6 h	[65]
	Beta-tubulin	N	Growth in mouse lung alveoli for 4 h or 14 h	[66]
	Beta-tubulin *	N	Four formed fungi balls or 2 mL of fungal suspension in NaCl incubated for 3 h at 25, 30, 35 and 40 °C	[67]
	Beta-tubulin *	N	Treatment with sub-lethal and lethal amphotericin B (AmB) concentrations in Sabouraud medium for 24 h at 37 °C with shaking	[68]
	Beta-tubulin	N	Treatment with 4 mg/L itraconazole or dimethyl sulfoxide in Vogel’s medium at 37 °C with shaking at 250 rpm for 14 to 16 h	[69]
	Beta-tubulin	N	Growth in mouse lungs subcutaneously injected with 40 mg/kg Kenalog 1 day, or intraperitoneally with 175 mg/kg of cyclophosphamide 2 days prior to inoculation	[70]
	*tefA*			
	GAPDH	N	Exposure of *A. fumigatus* to human airway epithelial cells or human bronchial epithelial cells	[31]
	GAPDH	N	Treatment with 10 ng/mL of itraconazole (CEA17) or 5 μg/mL of itraconazole (mutant strains) in YG medium supplemented with 1.2 g/L of uracil and uridine for 24 h at 37 °C	[71]
	Putative 1,3-beta-glucan synthase catalytic subunit	N	Barrat’s minimal nitrate medium in the presence or absence of oxidative stress and/or iron-limitation for 33 h and 50 h	[72]
	*TEF1*	N	*in vitro*: (1) YPD media for 4 h, 8 h and 1 to 7 days at 37 °C, (2) YPD media for 24 h at 37 °C (3) YPD or RPMI medium for 24 h and 72 h at 37 °C, (4) YPD medium for 5 to 8 days at 37 °C *in vivo:* (1) Mouse lung incubation for 4 h, 8 h, and 1 to 7 days, (2) mouse lung incubation for 24 h and 72 h, (3) mouse lung incubation for 5 to 8 days	[73]
	*TEF1*	N	Growth in a glucose (3%)-yeast extract (YE; 1%) liquid medium at 37 °C for 16 h	[74]
*A. luchuensis*	Actin	N	Growth on rice as rice koji and sampling at the stages in the process of shimaishigoto (40 °C, 30 h) and dekoji (40 h)	[26]
	Actin	N	Citric acid production (CAP) medium at 30 °C for 0 h or 12 h with shaking at 163 rpm	[75]
*A. nidulans*	Actin	N	Low or high inorganic phosphate yeast extract medium (YEM) and MM at 37 °C for 17 h with shaking at 200 rpm	[76]
	Beta-tubulin			
	Actin	Y	Yeast extract-agar-glucose (YAG) supplemented with 15% polyethylene glycol (PEG) (*w/v*) compared to YAG without supplementation for 2 days	[77]
	Actin	N	Complete medium (CM) or MM with inducing or non-inducing supplements at 28 °C for 16 h with 250 rpm shaking	[78]
	Actin	N	YAG supplemented with 5 mM uridine and 10 mM uracil (YUU) at 37 °C for 24 h	[79]
	Actin	Y	MM supplemented with 0.1% fructose and 5 mM urea at 30 °C for 15 h with shaking at 150 rpm	[30]
	GAPDH			
	Beta-tubulin	N	YG medium in the presence or absence of drugs (camptothecin, imazalil, itraconazole, hygromycin, and 4-nitroquinoline oxide) for 8 h	[80]
	Beta-tubulin	N	3% lactose MM for 18 h, after which a source of induction and/or repression was added, and cultures incubated for 4 h	[81]
	Beta-tubulin	N	MM with exposure to light or darkness at 23 °C for 24 h (spores) or 30 °C for 40 h in distilled water (conidia)	[82]
	Histone 2B			
	Beta-tubulin *	N	MM supplemented with 50 mM ethyl methyl ketone or 1% glucose at 37 °C for 16 h to 18 h with shaking at 150 rpm	[83]
	EEF-3 Elongation Factor	N	Glucose-free minimal nitrate medium or minimal nitrate medium at 37 °C for 4 h or 24 h	[84]
	NAPDH	N	Modified MMPKRUU medium under K sufficient or deficient conditions at 37 °C for 24 h with shaking at 220 rpm	[85]
	Putative ribosomal protein L37	Y	Liquid MM under standard conditions	[86]
	Putative ribosomal protein L3			
*A. niger*	18S rRNA	N	PDA supplemented with different carbon and nitrogen sources	[87]
	18S rRNA *	N	Subculturing of transformants in the presence of 1.25 mg/mL of 5-fluoroorotic acid with uridine and uracil	[88]
	Actin	N	Liquid AMM for 0, 3, 6, 12 and 72 h and on AMM plates for 5 days	[89]
	Actin	N	Liquefied corn starch medium at 35 °C for 72 h with shaking at 330 rpm	[90]
	GAPDH			
	Actin	N	CM for 25 h	[91]
	GAPDH	N	Glucose maltose polypeptone yeast extract (GMPY) media at 30 °C and 250 rpm for 2 days	[92]
	Histone H2B	N	MM supplemented with L-rhamnose or L-rhamnonate at 30 °C with shaking at 250 rpm	[93]
	Histone-encoding Gene	Y	Growth in bioreactors on sorbitol as the carbon source with 1 mM D-xylose or 50 mM D-xylose	[94]
*A. nomius*	Calmodulin	N	Coconut agar at 25, 30 and 35 °C for 7 days	[95]
*A. oryzae*	18S rRNA *	N	Subculturing of transformants in the presence of 1.25 mg/mL of 5-fluoroorotic acid with uridine and uracil	[88]
	Actin	N	MM whole culture broth grown on a cellulose nitrate filter for 42 to 48 h until pigmented conidiospores present	[96]
	Actin	N	DPY agar medium for 48 h	[25]
	Actin	N	PDA medium at 30 °C for 7 days	[97]
	Beta-tubulin	N	Modified Czapek–Dox (CD) minimal agar at 30 °C for 48 h with 200 rpm shaking	[98]
	Beta-tubulin *	N	(1) α-amylase transcripts: Inducing (1% sorbitol plus 1% starch), non-inducing (1% sorbitol), and repressing (1% sorbitol plus 1% starch plus 2% sucrose) conditions for 48 h (2) glucoamylase A transcripts: Inducing conditions (in 1% starch) or repressing conditions (in 2% sucrose) at 30 °C for 24 h	[83]
	Histone H1	N	Growth on 5 g of wheat bran containing 2, 3, 4, 6 or 8 mL of water at 30 °C for 48 h	[99]
	Histone H1	N	MM at 30 °C for 5 days	[100]
	Histone H2A	N	DPY liquid medium at 30 °C for 2 days	[101]
*A. parasiticus*	18S rRNA *	N	Adye and Mateles (1964) medium for 48 h and 72 h	[102]
	18S rRNA	N	Stationary phase culture growth in PDB 30 °C for 4 days in the dark	[29]
	Actin	Y	Treatment with 0, 0.25, 0.5 or l.0 μg/mL aflastatin A for 1.5 to 3.5 days in PDB at 27 °C	[103]
	Actin	N	RPMI medium containing 25 or 50 mg/mL of vitamin C for 3 days at 28 °C	[104]
	Actin	Y	Co-incubation with *Kluyveromyces lactis* at 30 °C for 48 h	[105]
	Beta-tubulin *	N	YES or YEP media at 28 °C for 4 days	[48]
	Beta-tubulin	N	Yeast extract sucrose peptone (YESP) medium modified with 1% sodium nitrate (YESP-N) or 1% tryptophan (YESP-T) at 10, 15, 25, 30 and 37 °C for 96 h with shaking at 100 rpm	[106]
	Beta-tubulin *	N	Treatment or no treatment with the subinhibitory concentrations of carvacrol or trans-cinnamaldehyde in PDB at 25 °C for 5 days	[50]
	Beta-tubulin *	Y	YES medium containing one of four antifungal peptides: PPD1, 66-10, 77-3, or D4E1	[55]
*A. sojae*	18S rRNA *	N	Adye and Mateles (1964) medium for 48 h and 72 h	[102]
*A. terreus*	Actin	Y	Lovastatin production media at 27 °C for 10 days with shaking at 220 rpm	[107]
	Actin	N	MID medium for 10 days after which 0.5, 1.0, 3.0 and 5.0% (*w/v*) of surface sterilised *Podocarpus. gracilior* leaves were added and incubated together for 20 days	[108]
	Beta-tubulin *	N	Four formed fungi balls or 2 mL of fungal suspension in NaCl incubated for 3 h at 25, 30, 35 and 40 °C	[67]
	Beta-tubulin *	N	Treatment with sublethal and lethal concentrations of AmB in Sabouraud media at 37 °C for 24 h with slight shaking	[68]
*A. westerdijkiae*	Beta-tubulin	N	CYA in the presence or absence of *Debaryomyces hansenii* at 28 °C for 3 and 7 days	[109]
	GAPDH	N	YES medium at 25 °C for 96 h	[110]

Y, validation was provided; *, analysed more than one species of *Aspergillus* and appear twice in the table.

**Table 2:** Recommended reference genes for specific species and experimental conditions based on experimental validation.

Species	Reference Gene	Forward Primer (5′-3′)	Reverse Primer (5′-3′)	Experimental Conditions	Ref.
*A. aculeatus*	GAPDH	TACCGCTGCCCAGAACATCA	GGAGTGGCTGTCACCGTTCA	Minimal media (MM) with 1% (*w/v*) polypeptone, 1% (*w/v*) glucose, 1% (*w/v*) avicel, 1% (*w/v*) xylose, or 1% (*w/v*) arabinose for 3 h or 6 h	[35]
*A. carbonarius*	Ubiquitin-conjugating Enzyme	CCGAAGGTCAACTTCACCAC	GGCATATTTGCGAGTCCATT	MM at 25 °C, without shaking (ochratoxin A (OTA)-inducing) conditions, for 4, 6 and 8 days in the dark	[32]
*A. fischeri*	Beta-tubulin	GCTCTTCCGTCCCGATAACTT	CCTTGGCCCAGTTGTTACCA	Growth on a hydrophobic polyvinylidene fluoride (PVDF) membrane on top of oatmeal agar for 3, 6 or 30 days (wildtype only)	[38]
	Histone 3	CAAGAAGCCTCACCGCTACAAG	GACTTCTGGTAGCGACGGATTT		
*A. flavus*	18S rRNA	GCAAATTACCCAATCCCGACAC	GAATTACCGCGGCTGCTG	Co-culture with *Listeria monocytogenes* in malt extract broth (MEB) at 25 °C and 30 °C for 7 days	[41]
	Beta-tubulin	CGCATGAACGTCTACTTCAACGAG	AGTTGTTACCAGCAGCGGACT		
	Calmodulin	CTTCCCCGAATTCCTTACC	TCACGGATCATCTCATCGAC		
	Beta-tubulin	CTTGTTGACCAGGTTGTCGAT *	GTCGCAGCCCTCAGCCT*	Inoculated onto 25 g of wheat and grown at 30 °C in open petri dishes with wetted filter paper for 9 days	[46]
	Beta-tubulin	AACGTCTACTTCAACGAGGCCA	GTACCAGGCTCAAGATCAACGAG	Malt extract agar (MEA) media supplemented with 0.5 mM eugenol for 4 days at 27 °C in the dark	[52]
	GAPDH	CGTGTTGTTGACCTCATTGCCT	GGTGACCTGATAATCCGGGAAC		
	Beta-tubulin	AACGTCTACTTCAACGAGGCCA	GTACCAGGCTCAAGATCAACGAG	Co-incubation with *Streptomyces roseolus* on International Streptomyces Project-2 (ISP2) medium at 30 °C for 4 days at a relative humidity of 80% in a Vötsch chamber	[54]
	GAPDH	CGTGTTGTTGACCTCATTGCCT	GGTGACCTGATAATCCGGGAAC		
	Beta-tubulin	TCTCCAAGATCCGTGAGGAG	TTCAGGTCACCGTAAGAGGG	Yeast extract sucrose (YES) medium containing one of four antifungal peptides: PPD1, 66-10, 77-3, or D4E1	[55]
	Cytochrome C oxidase Subunit V	CGTCATTCACTTGTTCGCTAAG	CCTTGGCATACTCGTTGGAAG	Sucrose low salts (SLS), SLS supplemented with 117 mM ammonium sulphate, and lactose low salts, at acidic or alkaline pH	[111]
	Histone H4	TCGTCGTGGTGGTGTCAAG	TTGGCGTGCTCAGTGTAGG		
*A. fumigatus*	18S rRNA	TCTTGTTAAACCCTGTCGTGCTGG	GTGTACAAAGGGCAGGGACGTAAT	Treatment with 125 μg/mL artemisinin or solvent for 3 h in Roswell Park Memorial Institute (RPMI) 1640 medium at 37 °C	[27]
	Actin	TGCCCTTGCTCCCTCGTCTA	ACCGCTCTCGTCGTACTCCT	Mandels’ salt solution with 1% oat spelts xylan for 0, 2, 4, 6 and 17 h	[16]
	Histone H4	GCTCGTCGTGGTGGTGTCAA	TGGCGTGCTCAGTGTAGGTG		
*A. nidulans*	Actin	TCAATCCCAAGTCCAACC (Tm 57 °C)	TACGACCGGAAGCATACA (Tm 57 °C)	Yeast extract-agar-glucose (YAG) supplemented with 15% polyethylene glycol (PEG) (*w/v*) compared to YAG without supplementation for 2 days	[77]
		AATGGTTCGGGTATGTGC (Tm 60 °C)	ACGCTTGGACTGTGCCTC (Tm 60 °C)		
	Actin-like protein	GTACGATGAGAGCGGTCCTT	CAGAAAATACGCGACAACGA	MM supplemented with 0.1% fructose and 5 mM urea at 30 °C for 15 h with shaking at 150 rpm	[30]
	GAPDH	CGACAACGAGTGGGGTTACT	GGCATCAACCTTGGAGATGT		
	Calmodulin	CCGAGTACAAGGAAGCTTTCTC	GAATCATCTCGTCGACTTCGTCGTCAGT	WB/MM supplemented with high, low or no iron for 24, 48 and 72 h	[144]
	Putative ribosomal protein L3	TTCCTCGCAAGACTCACAAG	TTGTGGTTGCAAGAGGTACG	Liquid MM under standard conditions	[86]
	Putative ribosomal protein L37	CGCCACAACAAAACTCACAC	TCTCGCTCCAGTTGTACTTGC		
*A. niger*	Actin	GGTCTGGAGAGCGGTGGTAT	cactgcGAAGAAGGAGCAAGAGCAGtG	*glaA*-inducing and -non-inducing conditions in modified Vogel-Medium with stir speeds and pH from 400–1000 per min and 3.0 to 5.5, respectively	[160]
	Cytochrome C oxidase Subunit V	GACCAAGGAGTGGCAGGAG	gaactgGGTGGGAGGCAGCAGtTC		
	Secretion Associated Binding Protein	gaacctACGGGTAAGGGCAAGGtTC	TCGCAACAATAAAGTCAACAGC		
	Histone-encoding Gene	ATCTTGCGTGACAACATCCA	CACCCTCAAGGAAGGTCTTG	Growth in bioreactors on sorbitol as the carbon source with 1 mM D-xylose or 50 mM D-xylose	[94]
*A. parasiticus*	Actin	CTTGACTTCGAGCAGGAGAT	TCTGGATACGGTCGGAGATA	Treatment with or without 1.0 μM of blasticidin A in potato dextrose broth (PDB) at 27 °C for 2 days	[140]
	Actin	CGCGGATACACCTTCTCCACTA *	ACGTAGCAGAGCTTCTCCTTGA *	Treatment with 0, 0.25, 0.5 or l.0 μg/mL aflastatin A for 1.5 to 3.5 days in PDB at 27 °C	[103]
	Actin	NA	NA	Co-incubation with *Kluyveromyces lactis* at 30 °C for 48 h	[105]
	Beta-tubulin	TCTCCAAGATCCGTGAGGAG	TTCAGGTCACCGTAAGAGGG	YES medium containing one of four antifungal peptides: PPD1, 66-10, 77-3, or D4E1	[55]
*A. terreus*	Actin	TCGTGACTTGACCGACTACC	TGATGTCACGGACGATTTCA	Lovastatin production media at 27 °C for 10 days with shaking at 220 rpm	[107]

*, used TaqMan system and therefore these primers have an associated probe, NA, not available.

## Supplementary Materials

The following are available online at https://www.mdpi.com/article/10.3390/genes12070960/s1, Table S1: Summary of experimental information for 90 RT-qPCR studies of *Aspergillus*.
